# Proteomics for Studying the Effects of Ketogenic Diet Against Lithium Chloride/Pilocarpine Induced Epilepsy in Rats

**DOI:** 10.3389/fnins.2020.562853

**Published:** 2020-09-29

**Authors:** Yu-qin Zheng, Mei-fang Jin, Gui-hai Suo, You-jia Wu, Yu-xiao Sun, Hong Ni

**Affiliations:** ^1^Division of Brain Science, Institute of Pediatric Research, Children’s Hospital of Soochow University, Suzhou, China; ^2^Department of Pediatrics, Affiliated Hospital of Nantong University, Nantong, China

**Keywords:** ketogenic diet, antiepileptogenic, proteomics, hippocampus, rat-brain

## Abstract

The ketogenic diet (KD) demonstrates antiepileptogenic and neuroprotective efficacy, but the precise mechanisms are unclear. Here we explored the mechanism through systematic proteomics analysis of the lithium chloride-pilocarpine rat model. Sprague-Dawley rats (postnatal day 21, P21) were randomly divided into control (Ctr), seizure (SE), and KD treatment after seizure (SE + KD) groups. Tandem mass tag (TMT) labeling and liquid chromatography-tandem mass spectroscopy (LC-MS/MS) were utilized to assess changes in protein abundance in the hippocampus. A total of 5,564 proteins were identified, of which 110 showed a significant change in abundance between the SE and Ctr groups (18 upregulated and 92 downregulated), 278 between SE + KD and SE groups (218 upregulated and 60 downregulated), and 180 between Ctr and SE + KD groups (121 upregulated and 59 downregulated) (all *p* < 0.05). Seventy-nine proteins showing a significant change in abundance between SE and Ctr groups were reciprocally regulated in the SD + KD group compared to the SE group (i.e., the seizure-induced change was reversed by KD). Of these, five (dystrobrevin, centromere protein V, oxysterol-binding protein, tetraspanin-2, and progesterone receptor membrane component 2) were verified by parallel reaction monitoring. Kyoto Encyclopedia of Genes and Genomes (KEGG) pathway analysis indicated that proteins of the synaptic vesicle cycle pathway were enriched both among proteins differing in abundance between SE and Ctr groups as well as between SE + KD and SE groups. This comprehensive proteomics analyze of KD-treated epilepsy by quantitative proteomics revealed novel molecular mechanisms of KD antiepileptogenic efficacy and potential treatment targets.

## Introduction

Epilepsy is a chronic disease characterized clinically by recurrent and unpredictable seizures (Fisher et al., 2005) due to uncontrolled neuronal hyperactivity. A recent large-scale epidemiological survey of 196 countries and regions around the world found that there were 45.9 million people with epilepsy in 2016, with highest incidence in children aged 5 to 9 years (Beghi et al., 2019). Severe status epilepticus or recurrent seizures can cause cognitive decline, impair quality of life, and increase the risks of injury and sudden death (Nashef et al., 1995). The most common treatments for epilepsy are oral antiepileptic drugs (AEDs). However, about 30% of children are resistant to currently available AEDs (Pluta and Jablonski, 2011).

The ketogenic diet (KD) is a high fat, low carbohydrate regime widely considered an effective non-drug treatment for epilepsy with documented anticonvulsant, antiepileptogenic, and neuroprotective effects on clinically refractory epilepsy and animal models of epilepsy (Lusardi et al., 2015; Simeone et al., 2018; Karimzadeh et al., 2019). Multiple therapeutic mechanisms have been proposed for KD-induced antiepileptogenesis, including increased adenosine and decreased DNA methylation, reduced mTORC1 activity, and blockade of histone deacetylases (Koene et al., 2019; Boison and Rho, 2020). Thus, it is critical to comprehensively assess the molecular changes associated with the KD in epilepsy. Moreover, the KD is often unpalatable, especially to children, and must be sustained for years, resulting in poor compliance. In addition, constipation and weight loss are common adverse effects (Cai et al., 2017). In several clinical studies, the KD was also found to influence mood. Although most of these studies reported positive effects (Halyburton et al., 2007; McClernon et al., 2007; Dm et al., 2016), some reported no effects or even negative effects on mood (Lambrechts et al., 2013; Iacovides et al., 2019). These inconsistences may be related to the type of disease before KD treatment, the number of subjects, and the duration of KD compliance, necessitating larger-scale, multiple-center studies to assess the influence of the KD on mood in specific diseases. Death during KD treatment has also been reported secondary to severe infection and malnutrition (Kang et al., 2004; Suo et al., 2013). Therefore, a better understanding of the therapeutic mechanisms may improve clinical application and reveal new targets for clinical anti-epileptic treatment.

Previous studies on the antiepileptogenic efficacy of the KD focused mainly on changes in the expression of specific preselected proteins or genes, while few have used gene chips to objectively explore larger-scale gene expression changes associated with KD treatment of epilepsy (Bough et al., 2006; Jeong et al., 2010). Modern proteomics techniques can reveal similarities and differences in protein expression at the individual, pathway, and network levels under various physiological and pathological states, thus providing a more comprehensive understanding of disease pathology and progression (Atamna et al., 2002). Such proteomics studies have examined the pathogenesis of epilepsy (Walker et al., 2016; Sadeghi et al., 2017), but not the mechanisms underlying the antiepileptogenic action of KD. At present, the main technologies used in proteomics research are two-dimensional gel electrophoresis and mass spectrometry (MS). The former is technically demanding, is not amenable to automation, and has limited separation capacity, especially for low abundance and hydrophobic proteins. Alternatively, mass spectrometry is suitable for high-throughput analysis by automation and can discriminate proteins of similar size and isoelectric point.

Therefore, we conducted the first proteomics analysis of the antiepileptogenic response to KD in the rat lithium chloride-pilocarpine-induced epileptic model using MS-based tandem mass tag (TMT) quantitative proteomics.

## Materials and Methods

### Animals and Treatment

#### Animal Preparation

Postnatal day 21 (P21) Sprague-Dawley rats (*n* = 45) were obtained from JOINN Laboratories, Co. Ltd. (Suzhou, China) [License no. SCXK(SU) 2018-0006]. Animals were treated in accordance with the guidelines set by the National Institutes of Health (Bethesda, MD, United States) for the humane treatment of animals. Animal experiments were approved by the Animal Experimental Ethics Committee of Suzhou University. All rats were raised under a 12 h:12 h light: dark cycle with free access to drinking water and the indicated diet (normal or KD). Animals were protected from bright lights and excessive noise during housing. Rats were first randomly divided into a control group (Ctr, *n* = 10) and seizure model group (*n* = 35). Rats exhibiting status epilepticus following lithium chloride-pilocarpine treatment (detailed below) were then randomly assigned to the normal diet group (SE) or KD diet group (SE + KD).

#### Induction of Status Epilepticus

Status epilepticus was induced by lithium chloride-pilocarpine in accordance with our previous study (Chen et al., 2019). Briefly, 35 rats were injected intraperitoneally with 127 mg/kg lithium chloride (Sigma-Aldrich, United States) at P21 and 24 h later (P22) with 1 mg/kg scopolamine hydrobromide (TargetMol, United States) to reduce the peripheral cholinergic response to pilocarpine. Thirty minutes later, 320 mg/kg pilocarpine (Sigma-Aldrich, United States) was injected and response scored according to the Racine scale (Racine, 1972) as follows: (0) no abnormality; (1) mouth and facial movements; (2) head nodding; (3) unilateral forelimb clonus; (4) rearing with bilateral forelimb clonus; and (5) rearing and falling. Animals were selected for further study only if the seizure degree reached level IV or above (*n* = 28). The onset of status epilepticus was characterized by initial immobility and chewing followed by repetitive clonic activity of the trunk and limbs, repeated rearing with forelimb clonus and falling interspersed with immobility, chewing, and myoclonic jerks singularly or in series. Acute status epilepticus was stopped after 60 min by intraperitoneal administration of 300 mg/kg chloral hydrate (Sigma-Aldrich, United States). Five rats died due to generalized tonic seizures. Animals surviving status epilepticus were randomly divided into the normal diet SE group (*n* = 12) and SE + KD (*n* = 11) group. There were no differences in seizure duration and severity between groups. In each group, 10 rats were randomly labeled for weight and blood ketone measurements. After weight and blood ketone were measured, six rats in each group were randomly labeled for proteomics testing and parallel reaction monitoring (PRM) verification. Control group rats received the same treatments and evaluations but were injected intraperitoneally with 0.9% saline solution instead of pilocarpine. No epileptic seizures were observed in any Ctr group rat.

#### Dietary Intervention

During the modeling period (P21–P22), all groups were fed a normal diet. After vehicle treatment or status epilepticus induction, Ctr and SE groups continued to receive a normal diet for 28 days (4.5% fat, 20% protein and 50% carbohydrate), while the SE + KD group was fed the KD for 28 days (70% fat, 20% protein, and no carbohydrate). The KD formula was reported in detail previously (Ni et al., 2016). Further detail contents of the diets are shown in Table 1. Both diets were obtained from the Chinese Academy of Sciences, Shanghai Experimental Animal Center (Shanghai, China).

**TABLE 1:** Composition of normal and KDs.

**Component**	**Normal diet (%)**	**KD (%)**
Carbohydrate (starch)	50	−
Protein (casein)	20	20
Soybean oil	4.5	10
Lard	−	42
Butter	−	17
Sunflower seed oil	−	1
Fiber	5	5.5
AIN-76 mineral mixture	8	3.5
AIN-76 vitamin mixture	2.3	1
Energetic (kcal/g)	4	7.1
Ketogenic ratio	0.37:1	3.5:1

### Weight and Blood Ketone Monitoring

Body weight and blood ketones were recorded at P49. After the rats were anesthetized, blood samples were collected from the tail vein and blood ketone levels measured using a Keto-detector (Beijing Yicheng Bioelectronics Technology, Co., Ltd., China).

### Protein Extraction and Digestion

Samples of hippocampus were extracted, flash frozen to −80°C, ground into powder over liquid nitrogen, and transferred to 5-mL centrifuge tubes. Four volumes of pyrolysis buffer containing 8M urea and 1% protease inhibitor mixture (Calbiochem, San Diego, CA, United States) were added and the mixture sonicated three times on ice at high intensity using a Scientz ultrasonic system (Scientz, Ningbo, China). After centrifugation at 12,000 × *g* for 10 min at 4°C, the supernatant was transferred into another centrifuge tube, and the sediment at the bottom was discarded. The supernatant protein concentration was measured by the BCA kit (Beyotime, China). Supernatant proteins were then digested in trypsin (Promega, Madison, WI, United States) as described (Chen et al., 2018).

### Tandem Mass Tag (TMT) Labeling

After protein digestion, peptides were desalinated on a chromatographic X C18 SPE column (Phenomenex, Torrance, CA, United States), vacuum-dried, dissolved in 0.5M TEAB (Sigma-Aldrich), and labeled according to the operation instructions of the 9-plex TMT kit (Thermo Fisher Scientific).

### High-Performance Liquid Chromatography (HPLC) Fractionation

Labeled peptides were fractionated into 60 samples over 60 min by high pH reverse-phase HPLC using an Agilent 300Extend C18 column (5 μm particles, 4.6 mm ID, 250 mm length) and 8–32% acetonitrile (pH 9.0) gradient. Peptides were combined into 14 fractions and dried by vacuum centrifugation for mass spectroscopy.

### Liquid Chromatography-Tandem Mass Spectroscopy (LC-MS/MS)

Peptides were dissolved in 0.1% formic acid (solvent A) and loaded directly onto a homemade reversed-phase analytical column (15-cm length, 75 μm inner diameter). Samples were then eluted at 350 nL/min using a mobile phase consisting of 0.1% formic acid in 98% acetonitrile solvent B under the control of an EASY-nLC 1000 UPLC system (Thermo Fisher Scientific). The elution protocol was as follows: 9–26% solvent B for 40 min, 26–35% solvent B for 14 min, 35–80% solvent B for 3 min, and holding at 80% for the last 3 min. Eluted peptides were then subjected to nanoelectrospray ionization (NSI) followed by tandem mass spectrometry (MS/MS) using the Q ExactiveTM Plus system (Thermo Fisher Scientific) coupled to the UPLC. The electrostatic voltage applied was 2.1 kV and the m/z scan range was 400 to 1500. Both intact peptides and fragments were detected in the Orbitrap at resolutions of 70,000 and 35,000 FWHM, respectively. Peptides were then selected for MS/MS using a normalized collision energy (NCE) setting of 28. A data-dependent procedure that alternated between one MS scan followed by 20 MS/MS scans was applied for the top 20 precursor ions above a threshold ion count of 1 × 10^4^ in the MS survey scan with 30.0 s dynamic exclusion. Automatic gain control (AGC) was set at 5E4. Fixed first mass was set as 100 m/z.

### Database Searches

The MS/MS data were processed using Maxquant (v.1.5.2.8) and searched against the Rat_Protemoe_1905 database (29,947 sequences). A reverse decoy database was used to calculate the false positive rate caused by random matching. Trypsin/P was specified as the cleavage enzyme allowing for up to two missing cleavages. The minimum peptide length was set at seven and the maximum number of peptide modifications at five. The mass tolerance for precursor ions was set to 20 ppm for the first search and to 5 ppm for the main search, and the mass tolerance for fragment ions was set as 0.02 Da. Carbamidomethyl on Cys was specified as the fixed modification, and acetylation and oxidation on Met were specified as variable modifications. False discovery rate (FDR) was adjusted to < 1%.

### Parallel Reaction Monitoring (PRM)

We used LC-PRMMS analysis to verify protein expression levels derived from TMT analysis. Peptides remaining from proteomics analyses (above) were dissolved in 0.1% formic acid (solvent A), loaded directly onto a homemade reversed-phase analytical column, and eluted at a constant flow rate of 500 nL/min using the following mobile phase protocol control by an EASY-nLC 1000 UPLC system (Thermo Fisher Scientific): 6 to 25% solvent B (0.1% formic acid in 98% acetonitrile) over 40 min, 25 to 35% solvent B over 12 min, 35 to 80% over 4 min, then holding at 80% for the last 4 min. The peptides were subjected to NSI followed by tandem mass spectrometry (MS/MS) using the Q ExactiveTM Plus system (Thermo Fisher Scientific) coupled to the UPLC. The electrospray voltage applied was 2.0 kV, m/z scan range was 360 to 1080 for full scan, and intact peptides were detected in the Orbitrap at a resolution of 70,000. Peptides were then selected for 20 MS/MS scans on the Orbitrap at a resolution of 17,500 using a data-independent procedure. AGC was set at 3E6 for full MS and 1E5 for MS/MS. The maximum injection time was set at 50 ms for full MS and 110 ms for MS/MS. The isolation window for MS/MS was set at 1.6 m/z. The NCE was 27% with high energy collision dissociation (HCD). The resulting MS data were processed using Skyline (v.3.6). Peptide settings were as follows: enzyme was set as trypsin [KR/P], max missed cleavage as 0, peptide length as 7–25, and fixed modification as alkylation on Cys. The transition settings were as follows: precursor charges were set as 2, 3, ion charges as 1, and ion as b, y. The product ions were set from ion 3 to last ion, and the ion match tolerance was set as 0.02 Da.

### Bioinformatics Analysis

#### Gene Ontology (GO) Annotation

Gene Ontology is a major bioinformatics initiative to unify gene and gene product attributes across all species. The GO annotations for this study were derived from the UniProt-GOA database^1^. First, identified protein IDs were converted to UniProt IDs and then mapped to GO IDs. For identified proteins not annotated by the UniProt-GOA database, InterProScan was used to annotate GO function based on protein sequence alignment. Proteins were classified by GO annotation based on three categories: biological process, cellular component, and molecular function.

#### Kyoto Encyclopedia of Genes and Genomes (KEGG) Pathway Annotation

Proteins were then annotated to KEGG pathways using the online service tools KEGG automatic annotation server (KAAS) and KEGG Mapper.

#### GO and KEGG Pathway Functional Enrichment

A two-tailed Fisher’s exact test was used to test the enrichment of identified proteins against all proteins in GO and KEGG databases, with a corrected *p* < 0.05 considered significant.

### Statistical Analysis

Body weights and blood ketones were compared among groups by one-way analysis of variance (ANOVA) with the indicated *post hoc* tests for pair-wise comparisons. A *p* < 0.05 was considered significant for all tests. GraphPad Prism version 5.0 was used for all data processing.

## Results

### Effects of the Ketogenic Diet on Appearance

**FIGURE 1:**
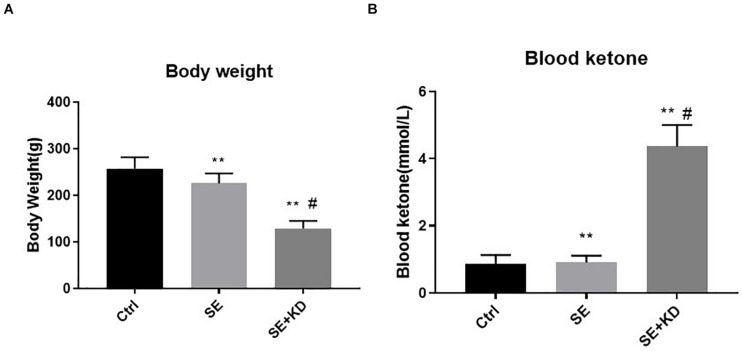
Comparison of body weight **(A)** and blood ketones **(B)** among control (Ctr), seizure (SE), and seizure with ketogenic diet (SE + KD) groups at P49 (*n* = 10 rats/group). Body weights were significantly reduced in SE and SE + KD groups compared to the Ctr group, and significantly lower in the SE + KD group compared to the SE group. Blood ketone level was significantly higher in the SE + KD group compared to Ctr and SE groups, but did not differ between Ctr and SE groups. **p < 0.01compared to Ctr group, #*p* < 0.01 compared with SE group.

Rats receiving the KD diet following status epilepticus induction (SE + KD group) gained substantially less weight after the 28 days observation period than both seizure-induced rats fed a regular diet (SE group, *p* < 0.01) and control rats (Ctr group, *p* < 0.01) (Figure 1A). Most SE + KD rats developed constipation and oily fur but otherwise were active and showed no evidence of infectious or respiratory complications, and none of them died.

### Blood Ketones

As shown in Figure 1B, blood ketone levels were significantly higher in the SE + KD group than Ctr and SE groups (*p* < 0.01), but did not differ between Ctr and SE groups (*p* > 0.05).

### LC-MS/MS

**FIGURE 2:**
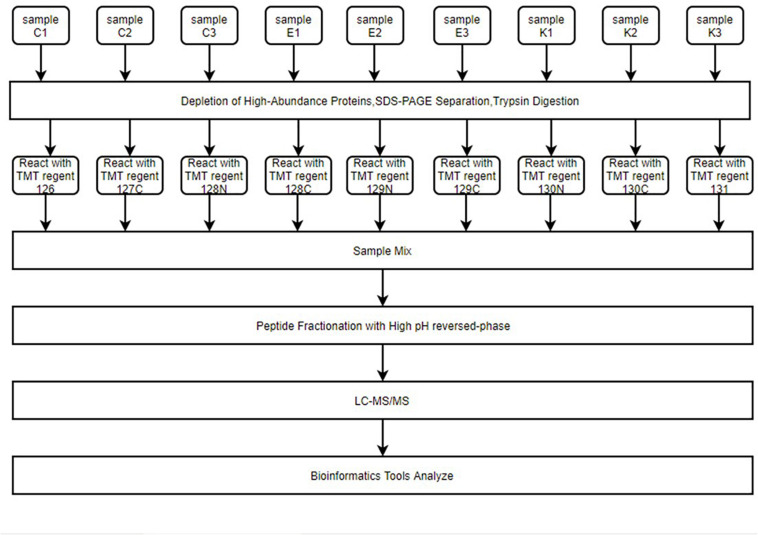
The entire proteomics experimental process. Hippocampus samples were reacted with different isotope-labeling TMT regents after immunoaffinity depletion of high-abundance plasma proteins, SDS-PAGE separation, and FASP digestion. Samples were mixed and peptides fractured by high pH reverse-phase chromatography. Finally, LC–MS/MS was used for high-throughput screening of samples. Peptides were then analyzed for function using multiple bioinformatics tools.

The abundances of hippocampal proteins were compared among Ctr, SE, and SE + KD groups using LC-MS/MS to identify those showing differential abundance caused by KD (Figure 2). A total of 238264.0 secondary spectrograms were obtained by mass spectrometry, and 82,100 spectrograms were available for analysis. A total of 41,645 peptide segments were identified, among which 38,097 were specific segments. In total, 5,564 proteins were identified, of which 4,740 were quantifiable. The screening criteria for differential abundance of proteins were fold-change > 1.2 (upregulated) or < 0.83 (downregulated) and *p* < 0.05. According to these criteria, 110 proteins exhibited a significant change in abundance between the SE and Ctr groups (18 upregulated and 92 downregulated), 180 between SE and SE + KD groups (121 upregulated and 59 downregulated), and 278 between SE + KD and Ctr groups (218 upregulated and 60 downregulated). Detailed data are provided in Supplementary Table S1. Optimized screening criteria were then applied for those proteins showing reciprocal abundance changes between SE vs. Ctr and SE + KD vs. SE groups. In total, 79 proteins met this condition (Supplementary Table S2), of which 72 were downregulated in the SE group compared to the Ctr group but upregulated in the SE + KD group compared to the SE group (i.e., downregulation induced by seizure was reversed by KD). The five showing the largest fold-changes were Hmgb3 protein, cyclic nucleotide-gated channel beta 3, aldose reductase-related protein 1-like, complexin 3, and solute carrier family 17 (sodium-dependent inorganic phosphate cotransporter) member 6. The other seven proteins showing reciprocal regulation were upregulated in the SE group compared to the Ctr group but downregulated in the SE + KD group compared to the SE group. The five proteins showing the largest fold changes among these seven were round spermatid basic protein 1, uncharacterized protein M0R9L6, cyclin dependent kinase inhibitor, reproductive homeobox on X chromosome 12, and IQ motif containing GTPase activating protein 1 (Predicted) isoform CRA.b.

### PRM Verification

To further verify the results of MS, five of these 79 reciprocally regulated proteins (dystrobrevin, centromere protein V, oxysterol-binding protein, tetraspanin-2, and progesterone receptor membrane component 2) were selected for PRM analysis. The screening criteria for PRM were based on the following principles: (1) proteins with potential biological function and significance; (2) proteins with a peptide fragment of no less than 1; (3) proteins associated with epilepsy but not reported or reported in only a few previous proteomic studies. Seven target peptide fragments of these five proteins were analyzed by Skyline, and the distributions of fragment ion peak areas are presented in Supplementary Figures S3–S9. The mean relative abundances of the target peptide fragments in each sample group are shown in Table 2. Differences in abundance of relative target proteins among sample groups were further calculated based on abundance of the corresponding peptide fragment (detailed data are provided in Table 3). Quantitative information on target peptide fragments was obtained from all nine samples. Compared to the Ctr group, the abundances of dystrobrevin, centromere protein V, oxysterol-binding protein, tetraspanin-2, and progesterone receptor membrane component 2 were downregulated in the SE group but upregulated in the SE + KD group, consistent with TMT results.

**TABLE 2:** Quantitative analysis of target peptide abundance.

**Peptide**	**Protein accession**	**Protein**	**Protein gene**	**Ctr mean**	**SE mean**	**SE + KD_ mean**	**Ration SE/Ctr**	**Ration SE + KD/SE**	**Ration SE + KD/Ctr**
LAAESSSSQPTQQR	D4A772	Dystrobrevin	Dtna	0.97	0.92	1.11	0.95	1.21	1.14
SGASGGLSGGESR	D4A9A3	Centromere protein V	CenpV	1.08	0.73	1.19	0.68	1.63	1.1
SNPGGFGIAPHCLDEGTVR	D4A9A3	Centromere protein V	CenpV	0.95	1.05	1.01	1.11	0.96	1.06
ASNQSQPLER	Q5BK47	Oxysterol-binding protein	Osbpl2	0.90	0.80	1.30	0.89	1.63	1.44
ESSEQVQPTCPK	Q9JJW1	Tetraspanin-2	Tspan2	0.92	0.82	1.28	0.89	1.53	1.36
GGDGSPGGAGATAAR	Q5XIU9	Progesterone receptor membrane component 2	Pgrmc2	0.99	0.73	1.26	0.74	1.75	1.29
DFSLEQLR	Q5XIU9	Progesterone receptor membrane component 2	Pgrmc2	0.91	1.09	1.00	1.19	0.92	1.10

**TABLE 3:** Quantitative analysis of target protein abundance.

**Protein**	**Ctr mean**	**SE mean**	**SE + KD mean**	**SE/Ctr ratio**	**SE + KD/Ctr ratio**	**SE + KD/SE ratio**
Dystrobrevin	0.97	0.92	1.11	0.95	1.14	1.20
Centromere protein V	1.01	0.89	1.10	0.88	1.08	1.23
Oxysterol-binding protein	0.90	0.80	1.30	0.89	1.44	1.62
Tetraspanin-2	0.92	0.82	1.26	0.89	1.36	1.53
Progesterone receptor membrane component 2	0.95	0.91	1.14	0.95	1.19	1.25

### Bioinformatics Analysis

We use Bioinformatics tools to analyze the differential abundances of all proteins detected by MS.

#### GO Functional Annotation Analysis

**FIGURE 3:**
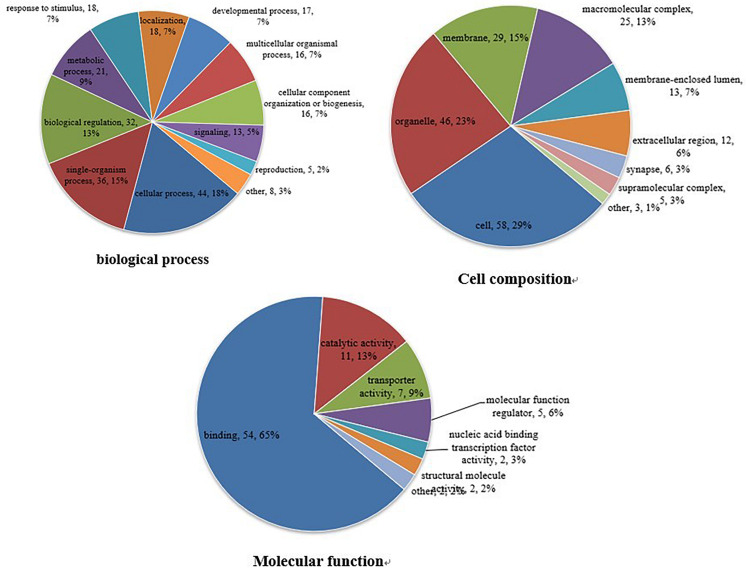
Gene ontogeny (GO) annotation. Differentially abundant proteins were annotated according to molecular function, cell composition, and biological process. Differentially abundant proteins are mainly annotated as ‘protein binding,’ ‘cell,’ and ‘cell process,’ respectively, in terms of molecular function, cell composition, and biological process.

The GO database is an international standardized functional classification system that comprehensively describes the characteristics of genes and their products. We performed GO functional annotation searches for all proteins identified in this study and then subjected those demonstrating differential abundance among groups to GO enrichment analysis using Fisher’s exact test. According to secondary GO annotations, most of the 79 reciprocally regulated proteins can be classified into three major categories: “molecular interactions,” “cell components,” and “biological processes.” The most common “molecular interaction” was “protein binding” (54 proteins, 65%), followed by “catalytic activity” (11 proteins), and “enzyme regulator” (seven proteins). The top three “cell components” classifications were “cell” (58 proteins), “organelle” (46 proteins), and “membrane” (29 proteins), while the top three “biological processes” classifications were “cellular process” (44 proteins), “single-organism process” (36 proteins), and “biological regulation” (32 proteins) (Figure 3). Additional classifications included “positive regulation of transferase activity,” “post-transcriptional regulation of gene expression,” “establishment of protein localization to organelle,” and “other important biological processes.” There were also significant group differences in expression of proteins with annotations “protein phosphatase binding,” “phosphatase binding,” “Ras GTPase binding,” “small GTPase binding,” “GTPase binding,” and “other molecular function” as well as “cytosol,” “macromolecular complex,” “nucleus,” “protein complex,” “vesicle,” and “other positioning proteins” (Supplementary Figure S1).

#### KEGG Pathway Analysis

**FIGURE 4:**
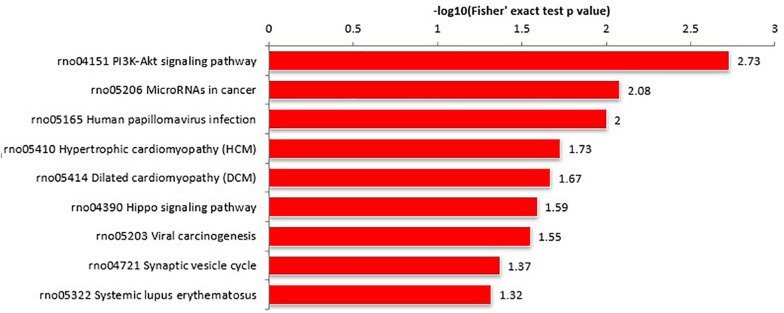
KEGG pathway enrichment analysis of proteins differing in abundance between SE and Ctr groups. The ‘PI3K-Akt signaling pathway’ showed highest enrichment. These differentially abundant proteins were also enriched in ‘synaptic vesicle cycle pathway.’

**FIGURE 5:**
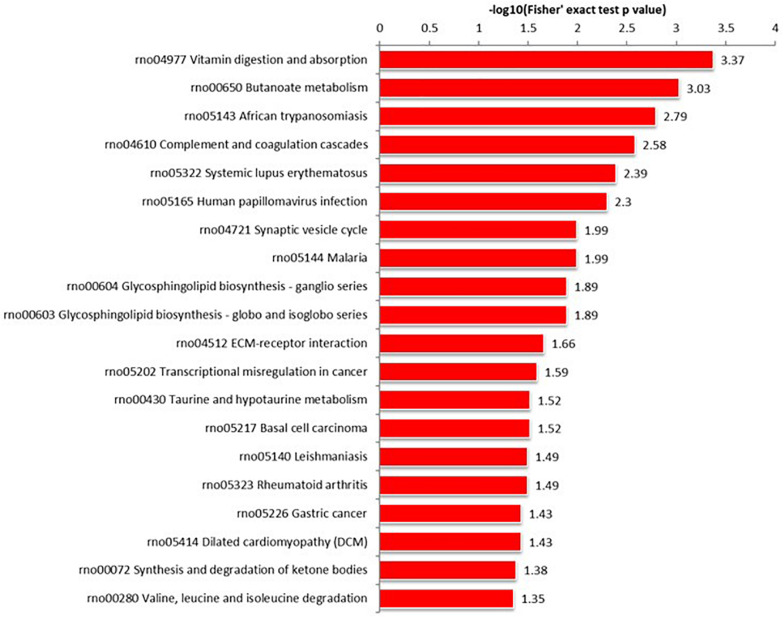
KEGG pathway enrichment analysis of proteins differing in abundance between SE + KD and SE groups. Vitamin digestion and absorption pathway showed highest enrichment. Differentially abundant proteins were also enriched in ‘synaptic vesicle cycle.’

Proteins interact within pathways and networks to perform specific biological functions and regulate pathophysiological processes. We used KEGG pathway analysis to reveal the biological pathways and relevant regulatory process involving hippocampal proteins differing in abundance among Ctr, SE, and SE + KD groups, especially those associated with epileptogenesis and the therapeutic mechanisms of KD. The proteins differing in abundance between SE and Ctr groups showed greatest enrichment in “PI3K-Akt signaling pathway,” proteins differing in abundance between SE + KD and SE groups showed greatest enrichment in “vitamin digestion and absorption pathway,” and proteins differing in abundance between SE + KD and Ctr groups showed greatest enrichment in “glycosaminoglycan degradation pathway” (Supplementary Figure S2). Proteins related to the synaptic vesicle cycle pathway were enriched not only among those differing in abundance between SE and Ctr groups but also among those differing in abundance between SE + KD and SE groups. Moreover, the abundances of complexin 3 and solute carrier family 17 (sodium-dependent inorganic phosphate cotransporter) member 6 in the synaptic vesicle cycle pathway were reduced in the SE group compared to the Ctr group, and downregulation of both proteins was reversed by the KD (Figures 4, 5 and Supplementary Tables S3, S4).

## Discussion

In recent years, our team has conducted a series of studies on the neuroprotective and antiepileptogenic efficacies of KD in rats. Tian et al. (2015, 2016) found that chronic KD treatment reversed the adverse neurobehavioral, cognitive, and neurochemical changes in Sprague-Dawley rats subjected to recurrent neonatal seizures. Moreover, these studies utilized a novel “twist” seizure model to assess both spontaneous and induced seizures by coupling early-life flurothyl-induced neonatal seizures with later penicillin exposure, and demonstrated that KD could also increase seizure threshold to penicillin. Lusardi et al. (2015) used two epileptic models to examine the effect of KD on epileptogenesis, and found that 100% of all normal-fed rats demonstrated stage-3 seizures or higher after 15 pentylenetetrazol injections, whereas only 37% of KD-fed rats reached comparable seizure stages. They also found that normal-fed animals exhibited spontaneous seizures of progressively greater severity and frequency following pilocarpine induction, whereas KD-fed animals showed a prolonged reduction in seizure severity and frequency. Collectively, these studies demonstrated that KD can suppress epileptogenesis in rats. These findings and those of our previous study provide theoretical and technical support for the antiepileptogenic and neuroprotective effects of KD.

In the current study, we identified 79 proteins that were reciprocally regulated by KD (i.e., exhibiting upregulation in the SE group compared to the control group but downregulation in the SE + KD group compared to the SE group or vice versa). These reciprocal changes may be attributed to the antiepileptogenic effect of the KD. Furthermore, the same reciprocal changes in five proteins (dystrobrevin, tetraspanin-2, oxysterol-binding protein, progesterone receptor membrane component 2, and centromere protein V) were verified by PRM. Proteins differing in abundance between both Ctr and SE groups as well as SE + KD and SE groups were enriched in synaptic vesicle recycling pathway proteins according to KEGG pathway analysis, and two of these proteins, solute carrier family 17 (sodium-dependent inorganic phosphate cotransporter) member 6 and complexin 3, were reciprocally regulated. We suggest the following pathogenic processes to explain epileptogenesis and mitigation by the KD. The blood–brain barrier (BBB) was initially damaged by lithium chloride-pilocarpine-induced SE as indicated by abnormal abundance of α-dystrobrevin (Rigau et al., 2007). In turn, BBB disruption induced neuroinflammation as evidenced by tetraspan-2 upregulation, which led to dysfunctional lipid metabolism as evidenced by oxysterol-binding protein upregulation. Dysfunction of lipid metabolism induced mitochondrial dysfunction and deficient autophagy as indicated by the changes in abundance of progesterone receptor membrane component 2 and centromere protein V, respectively. Finally, defective autophagy resulted in accumulation of damaged mitochondria, triggering epilepsy and neuronal death. Alternatively, each of these pathogenic processes was reversed by KD. In addition, KD upregulated the abundance of solute carrier family 17 (sodium-dependent inorganic phosphate cotransporter) member 6 and complexin 3, both of which are neuroprotective (Ono et al., 1998; Van Liefferinge et al., 2015).

The dystrobrevins (DBs) α-DB and β-DB are cytosolic proteins encoded by the DTNA and DTNB genes, respectively. Alpha-DB in astrocyte end-feet is an important regulator of BBB permeability. It was reported that the aquaporin-4 water channel and Kir4.1 potassium channel were downregulated in the brain of DTNA knockout mice, resulting in enhanced cerebral capillary permeability, gradual cerebral edema, and ultimate damage to neurovascular units (Lien et al., 2012). Damage to the BBB can induce astrocyte dysfunction, neuroinflammation, and epilepsy (Rempe et al., 2018; Swissa et al., 2019). Our results suggest that KD mitigates epilepsy development in part by restoring BBB function through increased α-DB abundance.

Tetraspan-2 (Tspan2) is a small transmembrane protein widely distributed in the central nervous system. Knockout of Tspan2 activates white matter astrocytes and microglia (de Monasterio-Schrader et al., 2013), suggesting that Tspan2 inhibits neuroinflammation, a central pathogenic process in epilepsy (Ngugi et al., 2013). During the development of epilepsy, astrocytes and microglia proliferate, activate, and release inflammatory factors, leading to abnormal neural network connections and aggravating neurotoxicity (Rana and Musto, 2018). In contrast, KD promotes neuroprotection and suppresses epileptogenesis by inhibiting this inflammatory response (Stafstrom and Rho, 2012; Simeone et al., 2018). In the current study, the abundance of Tspan2 was downregulated in the SE group compared to the Ctr group but upregulated after KD. Therefore, we speculate that KD also suppresses epileptogenesis by increasing Tspan2 and suppressing epilepsy-associated neuroinflammation.

There is a strong mutual interaction between cellular inflammation and lipid metabolism, as imbalanced lipid metabolism can result in inflammation (Sun et al., 2009), while inflammation can promote cellular lipid uptake and accumulation, and inhibit cholesterol efflux (Khovidhunkit et al., 2004; McGillicuddy et al., 2009). Oxysterol binding protein (Accession number: Q5BK47), also known as oxysterol binding protein-like 2 (OSBPL2), is a highly conserved transporter protein that controls cholesterol and PI (4,5) P2 levels in the plasma membrane (Wang et al., 2019b). In addition, OSBPL2 is involved in the synthesis of cholesterol and cholesterol ester. Knockout or silencing the OSBPL2 gene inhibited AMPK activity and increased intracellular cholesterol and cholesterol ester synthesis (Wang et al., 2019a; Zhang et al., 2019). Imbalanced cholesterol homeostasis is implicated in the pathogenesis of multiple disorders, including cardiovascular, cerebrovascular, and central nervous system diseases (Chistiakov et al., 2016; Xue-Shan et al., 2016; Puglisi and Yagci, 2019). Altered levels of cholesterol and certain oxysterols have been reported in the hippocampus of rats following kainic acid-induced epilepsy (Ong et al., 2003; Heverin et al., 2012). We found that levels of the lipid metabolism-related molecules ApoE, clusterin, and ACAT-1 were upregulated after flurothyl-induced recurrent seizures in neonatal rats, while KD reversed these changes as well as the cognitive and neurobehavioral abnormalities associated with seizures (Tian et al., 2015). Thus, KD may also protect against epilepsy and associated sequelae by normalizing lipid homeostasis. Indeed, the downregulation of OSBPL2 observed in the SE group compared to the Ctr group was reversed by KD, which may in turn reduce cellular cholesterol accumulation, thereby mitigating oxidative stress and mitochondrial damage (Wang et al., 2019a).

Accumulation of cholesterol is a major cause of mitochondrial dysfunction in different models and cells. In Alzheimer’s disease and Niemann-Pick type C disease, mitochondrial cholesterol accumulation disrupts membrane physical properties and restricts the transport of glutathione into mitochondrial matrix, thus impairing mitochondrial function (Torres et al., 2019). In a mouse non-alcoholic fatty liver disease model, cholesterol overload contributed to a reduction in mitochondrial membrane potential and ATP content, and to significant alterations in mitochondrial dynamics (Dominguez-Perez et al., 2019). Kim et al. (2006) found that 7-ketocholesterol enhanced 1-methyl-4-phenylpyridinium-induced mitochondrial dysfunction and cell death in PC12 cells. Progesterone receptor membrane component 2 (PGRMC2) is a member of the progesterone membrane-related receptor (MAPR) family. It contains a heme-binding domain similar to cytochrome EB5 and a recent study (Galmozzi et al., 2019) found that deletion of PGMRC2 reduced intracellular heme synthesis. Heme promotes neurogenesis as well as neuronal survival and growth. However, dysregulation of intracellular heme concentration can result in neurodegeneration and impaired neurological function (Gozzelino, 2016). Reduction of heme synthesis in primary rat hippocampal neurons using *n*-methyltropophyrin reduced mitochondrial complex IV, activated carbon monoxide synthetase, and altered amyloid precursor protein (APP)α and APPβ protein levels, suggesting that decreased heme contributes to the neuronal dysfunction of Alzheimer’s disease (Atamna et al., 2002). In accord with these findings, blockade of heme biosynthesis by siRNA-mediated knockdown and n-methyltropophyrin IX treatment in differentiated SH-SY5Y neuroblastoma cells resulted in mitochondrial membrane depolarization, lower intracellular ATP production, APP aggregation, suppressed soluble (s)APPα secretion, and increased sAPPβ secretion (Gatta et al., 2009). Reduced intracellular heme was shown to disrupt mitochondrial function. Moreover, mitochondrial dysfunction is a common pathway for neurodegeneration (Rusek et al., 2019), so we speculate that decreased abundance of PGRMC2 in the SE group compared to the Ctr group is indicative of mitochondrial dysfunction, consistent with our previous study showing that flurothyl-induced seizures significantly depolarized mitochondrial membrane potential, reduced mitochondrial fusion protein 2 expression, and upregulated dynamic related protein 1 (drp1) in hippocampus (Liu et al., 2018). Conversely, KD upregulated PGRMC2, suggesting that KD also protects against neuronal death and epilepsy by sustaining mitochondrial function (Simeone et al., 2018; Rusek et al., 2019).

Intracellular cholesterol accumulation not only damages mitochondria, but also impairs autophagy by interfering with the fusion of autophagosomes with endosomal-lysosomal vesicles (Barbero-Camps et al., 2018). Autophagy defects reduce the capacity of cells to remove damaged organelles, protein aggregates, macromolecules, and other toxic substances, leading to dysfunction and death. Further, numerous studies have implicated autophagy defects in epilepsy. Knockout of ATG-7, a key molecule in the autophagy cascade, leads to spontaneous seizures in mice, implying that inhibition of autophagy is sufficient to induce epilepsy (Boya et al., 2013). We also reported that ratio of LC3 II/I was downregulated in the hippocampus of newborn rats subjected to repeated seizure induction using flurothyl, indicating reduced numbers of autophagosomes, while p62 was upregulated, indicating enhanced autophagic flux (Ni et al., 2016). Centromere protein V (CENPV) contributes to the maintenance of cell dynamics by stabilizing microtubules (Honda et al., 2009), and this process is critical for autophagy. The microtubule organizing center (MTOC) containing CENPV is critical for centripetal transport of autophagosomes from the cell periphery as well as for the fusion of autophagosomes and lysosomes (Kochl et al., 2006; Xu et al., 2014). In the present study, the abundance of CENPV was reduced in the SE group, suggesting impaired microtubule stability leading to disrupted autophagy. We suggest that the ability of KD to activate autophagic pathways and reduce brain injury in response to both pentylenetetrazol-induced seizures (Wang et al., 2018) and lithium chloride–pilocarpine-induced seizures is mediated by CENPV upregulation.

The synaptic vesicle cycle plays an important role in maintaining the structural and functional integrity of the presynaptic terminal. Disruption of synaptic vesicle recycling leading to defects in synaptic transmission may contribute to neurological disorders such as Alzheimer’s disease and autism (Waites and Garner, 2011), and changes in synaptic vesicle recycling have also been observed in pilocarpine-induced status epilepticus model rats (Upreti et al., 2012). Further, KD can support synaptic vesicle recycling (Hrynevich et al., 2016), so we speculate that KD also prevents epileptogenesis by normalizing this pathway. In fact, synaptic vesicle recycling pathway proteins were enriched in both populations of proteins demonstrating differential abundance between groups (SE vs. Ctr and SE + KD vs. SE), and two proteins involved in the synaptic vesicle cycle, solute carrier family 17 member 6 and complexin 3, were reciprocally regulated (upregulated in the SE group and downregulated after KD). Thus, these proteins may be the targets of KD for preventing epileptogenesis.

Solute carrier family 17 (Sodium-dependent inorganic phosphate cotransporter), member 6, also known as vesicular glutamate transporter 2 (VGLUT2, encoded by Slc17a6) is a low affinity transporter of glutamate from the cytoplasm into synaptic vesicles (Bellocchio et al., 2000). Expression is lower in the hippocampus of patients with intractable epilepsy and hippocampal sclerosis (Van Liefferinge et al., 2015), consistent with findings of reduced abundance in the SE group. Lobo et al. (2011) found that high glutamic acid exposure reduced VGLUT2 expression by hippocampal neurons, resulting in substantial excitotoxicity. As KD reversed this decline, improved glutamate transport may also contribute to reduced epileptogenesis.

The complexins (Cplxs) are four small SNARE-related proteins (Cplx1–4) that regulate rapid calcium-triggered exocytosis of synaptic, and thus are important for maintaining synaptic neurotransmission (Hazell and Wang, 2005; Yi et al., 2006). Knockout of all Cplxs genes in mice significantly reduced the calcium-triggered release of glutamate and γ-aminobutyric acid from hippocampal and striatal neurons (Xue et al., 2008). Alternatively, injecting recombinant Cplx2 into Aplysia buccal ganglion neurons inhibited neurotransmitter release, while injecting Cplx2 antibody increased release (Ono et al., 1998). Mice harboring a mutant Cplx1 gene exhibited ataxia and sporadic convulsions (Reim et al., 2001). In the current study, the abundance of Cplx3 was decreased in the SE group and was restored by KD, suggesting that KD may mitigate epileptogenesis by reducing uncontrolled glutamate release, thereby restoring appropriate excitatory–inhibitory balance.

## Conclusion

To our knowledge, this is the first study to comprehensively analyze the changes in protein abundance induced by the KD diet among epileptic model rats through quantitative proteomics. We identified several 100 proteins demonstrating differential abundance among control, epilepsy, and epilepsy plus KD groups, of which 79 were reciprocally regulated by SE and KD. Five of these proteins were further verified by PRM. Subsets of these proteins are implicated in lipid metabolism, blood–brain barrier integrity, mitochondrial function, neuroinflammation, and autophagy. Other proteins regulated by both seizures and KD are involved in synaptic vesicle recycling. Collectively, these findings provide clues to the molecular mechanisms underlying the antiepileptogenic effects of KD and define multiple potential therapeutic targets. However, the precise molecular mechanisms of action require further verification. In future studies, we will focus on selected KD-sensitive target proteins and examine the phenotypic changes conferred by knockout and overexpression, identify proteins interacting with target proteins, observe the effects of target protein expression level changes on epilepsy-related pathophysiological processes, and examine if KD can preserve neural circuit integrity, normal behavior, and cognition in epileptic rats via changes in target protein expression.

## Data Availability Statement

The datasets presented in this study can be found in online repositories. The names of the repository/repositories and accession number(s) can be found in the article/ Supplementary Material.

## Ethics Statement

The animal study was reviewed and approved by Animal experiments were approved by the Animal Experimental Ethics Committee of Suzhou University.

## Author Contributions

HN designed the study. YZ and MJ performed the experiments. GS, YW, and YS analyzed the data and are responsible for the statistical analysis. YZ wrote the manuscript. All authors have reviewed and approved this version of the manuscript.

## Conflict of Interest

The authors declare that the research was conducted in the absence of any commercial or financial relationships that could be construed as a potential conflict of interest.

## Footnotes

1 http://www.ebi.ac.uk/GOA/

## Supplementary Material

Supplementary Figure 1 GO functional enrichment analysis of differentially abundant proteins.

Supplementary Figure 2 KEGG pathway enrichment analysis of proteins differing in abundance between SE + KD and Ctr groups.

Supplementary Figure 3 Distribution of fragment ion peak area: LAAESSSSQPTQQR (corresponding protein accession D4A772).

Supplementary Figure 4 Distribution of fragment ion peak area: ASNQSQPLER (corresponding protein accession Q5BK47).

Supplementary Figure 5 Distribution of fragment ion peak area: SGASGGLSGGESR (corresponding protein accession D4A9A3).

Supplementary Figure 6 Distribution of fragment ion peak area: SNPGGFGIAPHCLDEGTVR (corresponding protein accession D4A9A3).

Supplementary Figure 7 Distribution of fragment ion peak area: GGDGSPGGAGATAAR (corresponding protein accession Q5XIU9).

Supplementary Figure 8 Distribution of fragment ion peak area: DFSLEQLR (corresponding protein accession Q5XIU9).

Supplementary Figure 9 Distribution of fragment ion peak area: ESSEQVQPTCPK (corresponding protein accession Q9JJW1).

Supplementary Table 1 Differential abundance of proteins among Ctr, SE, and SE + KD groups.

Supplementary Table 2 Optimized differential abundance of proteins.

Supplementary Table 3 KEGG pathway enrichment analysis of proteins differing in abundance between SE and Ctr groups.

Supplementary Table 4 KEGG pathway enrichment analysis of proteins differing in abundance between SE + KD and SE groups.
