# Supplementary figures and images for: Proteomics for Studying the Effects of Ketogenic Diet Against Lithium Chloride/Pilocarpine Induced Epilepsy in Rats
Source: Front Neurosci. 2020 Sep 29;14:562853. doi: 10.3389/fnins.2020.562853 (PMC7550537; doi:10.3389/fnins.2020.562853)

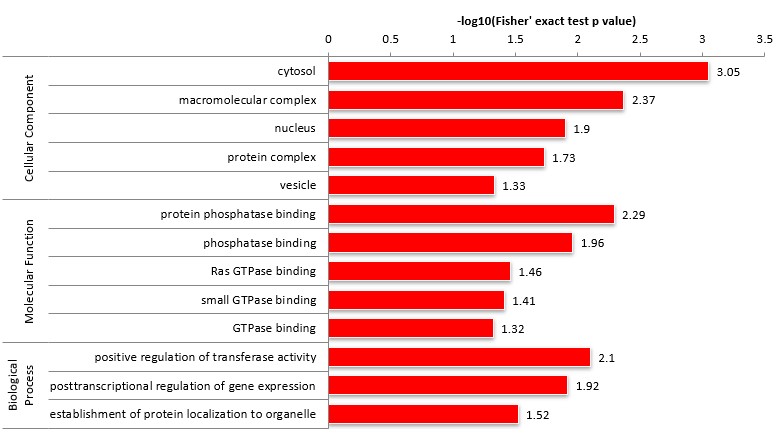

Supplement: Supplementary Figure 1 — GO functional enrichment analysis of differentially abundant proteins. [file Image_1.JPEG]

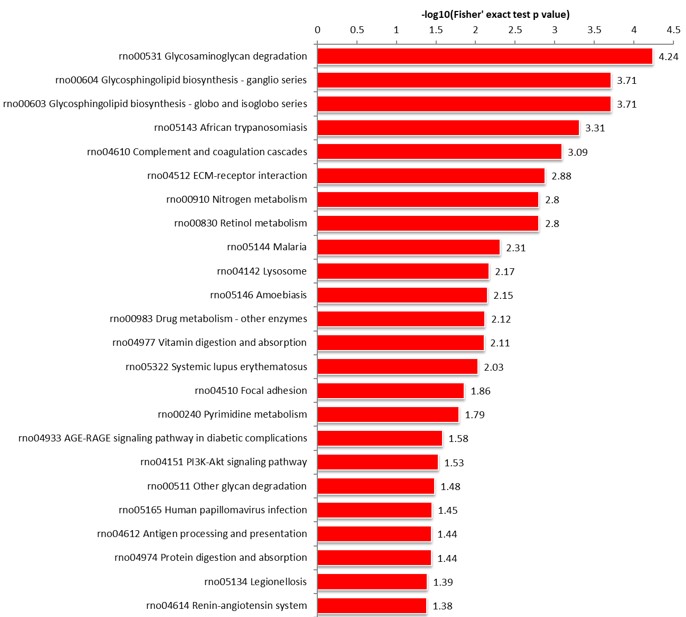

Supplement: Supplementary Figure 2 — KEGG pathway enrichment analysis of proteins differing in abundance between SE + KD and Ctr groups. [file Image_2.JPEG]

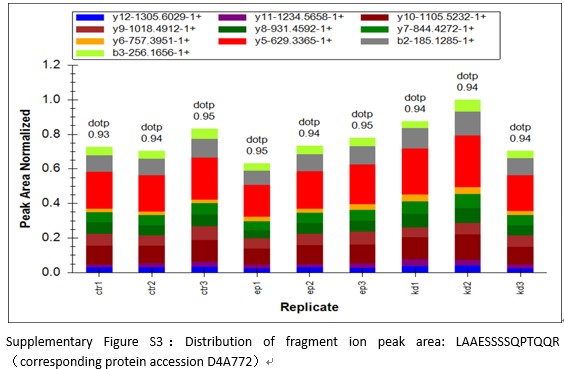

Supplement: Supplementary Figure 3 — Distribution of fragment ion peak area: LAAESSSSQPTQQR (corresponding protein accession D4A772). [file Image_3.JPEG]

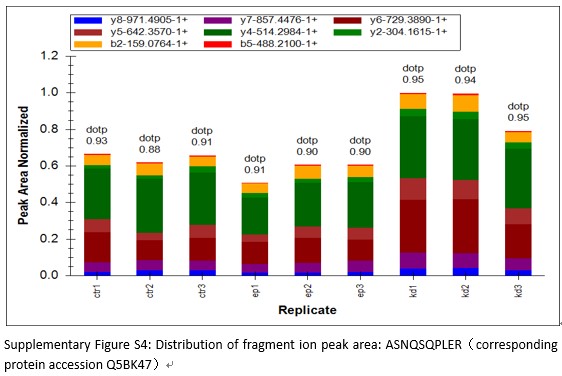

Supplement: Supplementary Figure 4 — Distribution of fragment ion peak area: ASNQSQPLER (corresponding protein accession Q5BK47). [file Image_4.JPEG]

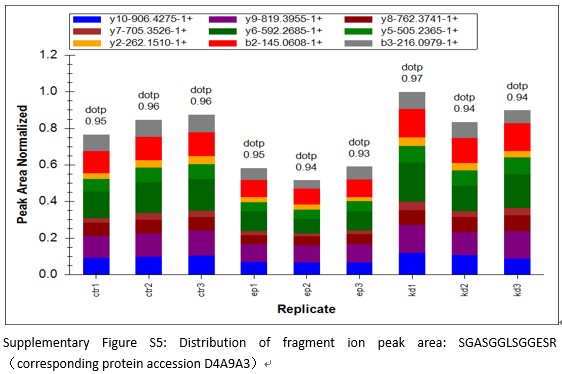

Supplement: Supplementary Figure 5 — Distribution of fragment ion peak area: SGASGGLSGGESR (corresponding protein accession D4A9A3). [file Image_5.JPEG]

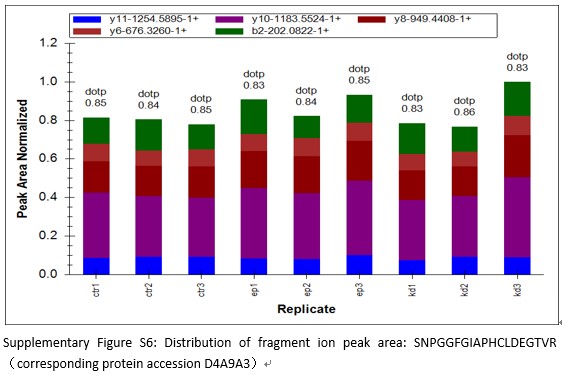

Supplement: Supplementary Figure 6 — Distribution of fragment ion peak area: SNPGGFGIAPHCLDEGTVR (corresponding protein accession D4A9A3). [file Image_6.JPEG]

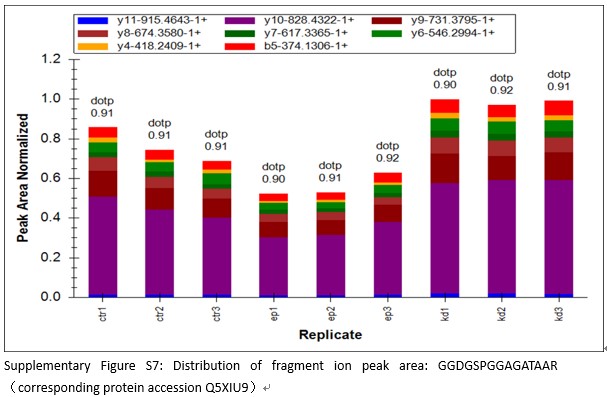

Supplement: Supplementary Figure 7 — Distribution of fragment ion peak area: GGDGSPGGAGATAAR (corresponding protein accession Q5XIU9). [file Image_7.JPEG]

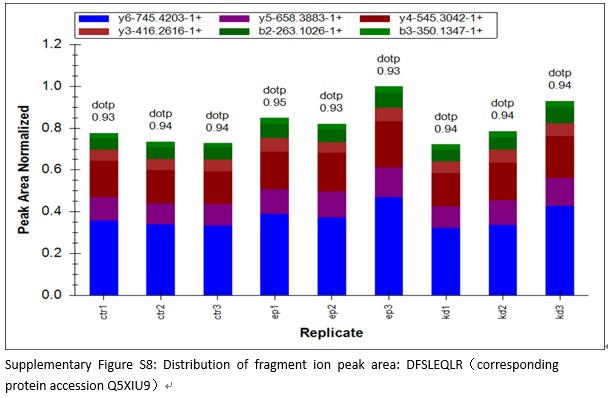

Supplement: Supplementary Figure 8 — Distribution of fragment ion peak area: DFSLEQLR (corresponding protein accession Q5XIU9). [file Image_8.JPEG]

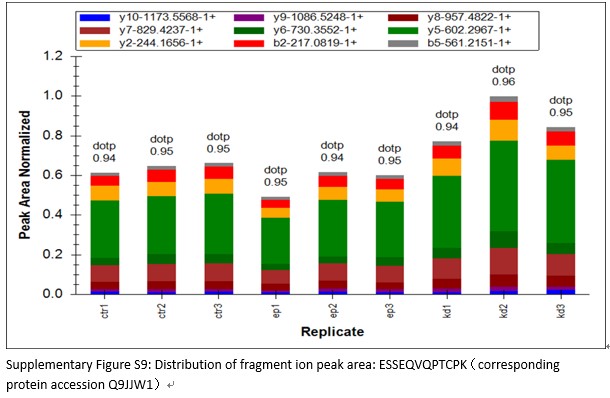

Supplement: Supplementary Figure 9 — Distribution of fragment ion peak area: ESSEQVQPTCPK (corresponding protein accession Q9JJW1). [file Image_9.JPEG]
